# Secondary Dyslipidemia In Obese Children - Is There Evidence For Pharmacological Treatment?

**DOI:** 10.5935/abc.20180187

**Published:** 2018-09

**Authors:** Ana Cristina Sayuri Tanaka

**Affiliations:** Unidade de Cardiologia Pediátrica e Cardiopatias Congênitas no Adulto - Instituto do Coração do Hospital das Clínicas da Faculdade de Medicina da Universidade de São Paulo, São Paulo, SP - Brazil

**Keywords:** Heart Defects, Congenital, Dyslipidemias, Oxidative Stress, Metabolic Syndrome, Indicators of Morbidity and Mortality

Obesity is a condition that has progressively increased throughout the world, also affecting children and adolescents, leading to high costs for health systems. Pediatric obesity is associated with dyslipidemia, oxidative stress, insulin resistance, and endothelial dysfunction, cardiovascular risk factors and components of the metabolic syndrome,^1^ and leads to adverse consequences such as early mortality and physical morbidity in adulthood in the short and long term.

Obesity-related dyslipidemia consists of increased triglycerides and free fatty acids, and decreased HDL-c (high-density cholesterol), normal or slightly increased LDL-c (low-density cholesterol), and increased VLDL-c (cholesterol of very low density). Plasma apolipoprotein B (apo B) concentrations are also frequently increased, in part due to increased hepatic production of apo B-containing lipoproteins.^2,3^

In most cases, dyslipidemia is a consequence of bad lifestyle habits, such as a diet rich in saturated or trans fats, and sedentarism. To plan monitoring and treatment, a cardiovascular risk stratification should be done since childhood, and not only the child, but especially the entire family living with him/her, should be educated. Longitudinal studies have shown that interventions in children are effective in the prevention of cardiovascular disease in adults.

The treatment of obesity-related dyslipidemia should be directed to weight loss through increased physical exercise and better eating habits, with a reduction in total calorie intake and reduced intake of essential fatty acids. Lifestyle changes synergistically improve insulin resistance and dyslipidemia.^4^ The child and the adolescent should be ideally followed by a nutritionist or nutrologist, because of the risk of growth and development impairment.

Interaction among genes, obesity and lipid levels, but also with the type of fat taken in the diet, was recently described.^5,6^ Studies suggest the potential utility of a nutrigenomic approach to dietary interventions to prevent or treat obesity and its associated dyslipidemia.^5,6^

Further studies should be conducted on the behavior of coronary artery disease markers, and of serum levels of total cholesterol, low-density lipoprotein, apolipoprotein B, and high-density lipoprotein in children and adolescents compared to adults,^2^ both in the pre- and post-treatment of obesity-related dyslipidemia, and in the short and long term, considering the cardiovascular risks, and the adverse effects resulting from pharmacological treatment, especially of statins.^3,7-11^

Lipid-lowering therapy should be started after at least six months of intensive lifestyle modification. The drugs used are statins, cholesterol absorption inhibitors (ezetimibe), bile acid sequestrants, phytosterol supplements, omega-3s, and fibrates.

Statins are the drugs of choice among all pharmacological agents to reduce LDL-c, non-HDL-c and/or apoB. However, statins do not lower triglycerides well, and do not completely correct the characteristic dyslipidemia observed in obesity, keeping a residual risk after therapy initiation.^11^ Recently, strategies for therapies combined with statins and other drugs to achieve even lower cholesterol levels have been reviewed.^11-15^

Children and adolescents with dyslipidemias who do not adequately respond to changes in lifestyle and habitual doses of lipid-lowering medications should be referred to specialist centers.

The work presented in this issue on secondary dyslipidemia in obese children demonstrates the scarcity of randomized clinical trials in the literature on the use of statins for the treatment of children and adolescents with obesity-related dyslipidemia.

Undoubtedly, this is a topic that should be investigated in depth and in details, with well-defined studies, to prove the efficacy of the several treatments already consecrated for the adult population in the pediatric and adolescent age.
